# Effect of a Multidisciplinary Outpatient Model of Care on Health Outcomes in Older Patients with Multimorbidity: A Retrospective Case Control Study

**DOI:** 10.1371/journal.pone.0161382

**Published:** 2016-08-18

**Authors:** Sepehr Shakib, Benjamin K. Dundon, John Maddison, Josephine Thomas, Melinda Stanners, Gillian E. Caughey, Robyn A. Clark

**Affiliations:** 1 Department of Clinical Pharmacology, Royal Adelaide Hospital, North Terrace, Adelaide, South Australia, 5000; 2 Discipline of Pharmacology, School of Medicine, University of Adelaide, North Terrace Adelaide, South Australia, 5000; 3 Monash Cardiovascular Research Centre, Monash Health, Victoria, South Australia 3000; 4 Clinical Education, School of Medicine, University of Adelaide, Adelaide, South Australia 5000; 5 School of Pharmacy and Medical Sciences, University of South Australia, Adelaide, South Australia, 5001; 6 Department of Nursing Acute Care & Cardiovascular Research, Flinders University of South Australia, Adelaide, South Australia, 5001; Azienda Ospedaliero Universitaria Careggi, ITALY

## Abstract

**Objective:**

To evaluate a holistic multidisciplinary outpatient model of care on hospital readmission, length of stay and mortality in older patients with multimorbidity following discharge from hospital.

**Design and Participants:**

A pilot case-control study between March 2006 and June 2009 of patients referred on discharge to a multidisciplinary, integrated outpatient model of care that includes outpatient follow-up, timely GP communication and dial-in service compared with usual care following discharge, within a metropolitan, tertiary referral, public teaching hospital. Controls were matched in a 4:1 ratio with cases for age, gender, index admission diagnosis and length of stay.

**Main outcome measures:**

Non-elective readmission rates, total readmission length of stay and overall survival.

**Results:**

A total of 252 cases and 1008 control patients were included in the study. Despite the patients referred to the multidisciplinary model of care had slightly more comorbid conditions, significantly higher total length of hospital stay in the previous 12 months and increased prevalence of diabetes and heart failure by comparison to those who received usual care, they had significantly improved survival (adjusted hazard ratio 0.70 95% CI 0.51–0.96, p = 0.029) and no excess in the number of hospitalisations observed.

**Conclusion:**

Following discharge from hospital, holistic multidisciplinary outpatient management is associated with improved survival in older patients with multimorbidity. The findings of this study warrant further examination in randomised and cost-effectiveness trials.

## Introduction

Many innovative multidisciplinary models of care have been developed for the treatment of individual chronic diseases, such as diabetes, chronic heart failure, chronic obstructive pulmonary disease and chronic kidney disease.[1–4] These structured models have been found to reduce fragmentation of information, improve continuity, coordination and the quality of patient care,[5,6] and importantly improve health outcomes including mortality and hospital readmission for patients with individual disease states.[4,7,8] However, over 65% of the older population will have multiple chronic conditions (multimorbidity).[9] Despite the high prevalence of multimorbidity and its negative impact on health outcomes and quality of life for patients and healthcare systems, there is limited evidence on appropriate models of care in this population.[10] Consequently, the development of care models capable of addressing the complexities of multimorbidity is of utmost importance in the pursuit of quality health care for this growing population.

For older patients with multimorbidity, care models need to include the holistic incorporation of disease-specific, guideline based recommendations that reconciles differences and conflicts between them, together with patient preferences and goals.[11] A recent systematic review examining models of care in primary and community settings for patients with multimorbidity reported improved medication management particularly if interventions were focused on functional or specific risk factors.[10] The majority of care models examined included some form of enhanced multidisciplinary team work and case management.[10] Only four of the ten studies included in the review focused on the outcomes of rehospitalisation or mortality, however the results were mixed.[12–14] Further a recent systematic review of hospital inpatient interdisciplinary team care interventions reported little effect on patient outcomes.[15] These were not specific to older patients with multimorbidity, the interventions did not include care coordination between hospital and the community setting and the traditional outcome measures assessed may not capture the complexities of high-quality health care delivery.[16]

Patients in primary care tend to be at lower risk of readmission, than those recently hospitalised and studies in this setting need to be larger and of longer duration to be able to demonstrate improvements in hospitalisation or mortality. Patients with multimorbidity who are discharged from hospital, have a high readmission rate, and their care can be more fragmented due to referrals to multiple subspecialty services on hospital discharge. Hence, multidisciplinary care models, which can demonstrate reductions in overall hospital readmission and patient mortality at the interface between the hospital and the community are needed to guide the optimal management of patients with multimorbidity. This is further supported by a 2015 Institute of Medicine (National Academy of Sciences, USA) report calling for well-designed studies to examine the impact of inter-professional education and collaborative multidisciplinary care on associations with patient and population health outcomes.[17]

Previously, we have examined the effect of a holistic multidisciplinary model of care in older chronic heart failure patients with comorbidity, based on multidisciplinary assessment and determination of individualised, reconciled evidence-based guideline recommendations on compliance with clinical guideline recommendations.[18] This model of care termed The Multidisciplinary Ambulatory Consulting Service (MACS) is delivered for patients with multiple chronic conditions, recently discharged from a metropolitan tertiary health care service. We demonstrated this model of care to be associated with high compliance to clinical guideline recommendations across all comorbid conditions present, including both non-pharmacological and pharmacological recommendations and health service utilisation[18]. We hypothesised that this model of care would decrease hospital readmission and mortality in a pilot study of older patients with multimorbidity after hospital discharge.

## Methods

Ethics approval for this study was obtained from the South Australian Government Royal Adelaide Hospital Human Research Ethics Committee. This study was deemed as a quality improvement activity, conducting an appropriate audit of a clinical service. In accord, written consent was not required as data which is routinely available within current clinical practice was used and analysed using non-identifiable data.

### Study Design, Setting and Participants

This pilot study was conducted at a large metropolitan, tertiary referral, public teaching hospital which is the major hospital within the Central Adelaide Local Health Network. We undertook a cumulative case-control study between 1 March 2006 and 1 June 2009 of patients with an index admission during this period that were discharged from any of the general medical units (index admission). Cases were those patients who following discharge were referred to the MACS service for outpatient management and the control group were those who received ‘usual care’ managed with standard, clinically driven follow-up by regular outpatient clinics and primary health care providers. Inclusion criteria for the cases included those with two or more chronic conditions, aged 65 years and older, who had at least two MACS clinic visits, and lived in the metropolitan area. Four controls were selected from the study source population from non-identifiable data, at the end of the study’s follow-up period after the identification of cases and if they met the study entry criteria. Based on the matching criteria (age, gender, index admission length of stay, and the first two letters of the ICD-10AM code for their index admission’s principal diagnosis), a minimum of six potential controls were randomly identified from the hospital’s administrative database (OACIS v7.1.0.104, Telus Health) as potential matches to each case. Final matching was based on a minimum of three out of four of the matching criteria and the control selection was blinded to any of the study outcomes. As we had a fixed number of cases over the study period (n = 252) we selected 4:1 controls / cases as this increased efficiency of the study, providing 80% power at 5% significance level to detect a difference of 10% between the cases and controls. MACS patients were excluded from analyses if they had uncommon principal diagnoses for which insufficient number of controls could be found.

Details of the MACS model of care have been published previously.[18] Briefly it includes holistic multidisciplinary assessment that includes patient preferences, evidence-based recommendations, medicines review by a pharmacist, outpatient follow-up, and dial-in service for advice or facilitated access to tertiary care. This is underpinned by regular structured general practitioner communication, and feedback regarding the patient’s management.

### Study Outcomes

Primary study outcomes included all non-elective readmission rates, total length of stay (LOS) for all readmissions during the follow-up period and mortality. Patients were followed from study entry (discharge date for control patients (usual care) or for the cases the date of the second MACS appointment) until death or study end (30 March 2010). Additional information, such as the patients’ coded comorbidities during the index admission, number of previous admissions and cumulative length of stay in the year prior to index admission, demographic information, and outcome data was extracted from the hospital database.

### Statistical Analysis

Frequency calculations, means, medians and interquartile range (IQR) were used to describe demographic and clinical characteristics for each group and differences between the groups were analysed by t-tests for continuous variables and chi-square statistics for categorical variables. Rates of readmission and total LOS for each group were compared using a negative binomial generalised estimating equation (GEE) with a 95% confidence interval (CI) to account for the matched design of the study. To compare the two groups while controlling for potential confounders, multivariate negative binomial GEEs were fitted to the data accounting for the duration of follow-up. Kaplan Meier analysis was used to examine survival between the MACS cases and controls and the log-rank test to compare the differences in survival between the two groups. Rates of survival between groups were examined using a Cox proportional hazards model (with robust variance estimation used to account for case-control matching). For both the Kaplan Meier and Cox proportional hazards analyses, survival duration was calculated using the discharge date in control patients, and the date of the second MACS appointment for MACS patients, in order to avoid immortal time bias. Covariates used in the Cox model included age, gender, marital status, country of birth, next of kin proximity, LOS of index admission, number of hospitalisations in the 12 months prior to index admission, primary diagnoses of primary index admission, comorbidities at index admission, discharge destination and duration of follow-up.

All analyses were performed using SAS Version 9.2 (SAS Institute, Cary, NC, USA)

## Results

A total of 252 MACS and 1008 usual care patients were included in the study. Overall the study cohort was predominantly older (median age 77 years old), equal gender representation, with a median of 4 days length of stay for the index admission (Table 1). Patients attending the MACS clinic were significantly more likely to be married, and less likely to be discharged to residential care. MACS patients had also spent more time in hospital in the year prior to the index admission (1.3 more days) and had a significantly greater number of chronic conditions. Diabetes and chronic heart failure were more common comorbid conditions amongst MACS patients, and dementia was more common in the usual care patients (Table 1). The total duration of follow up was 2545 patient years, with a median follow up of 22.3 months for MACS, and 24.7 months for usual care patients. The median number of clinic appointments for MACS patients during the follow up period was 4, with a range of 2–30.

**Table 1:** Patient demographic and clinical characteristics of study cohort.

Demographic and Clinical Characteristics	MACS (n = 252) N (%)	Usual care (Control) (n = 1008) N (%)
Matching Variables		
Age (median and IQR)^1^	77.0 (65.3–82.0)	77.0 (66.0–82.0)
Male^1^	127 (50.4%)	506 (50.2%)
Length of stay of index admission (days)^1^ (median and IQR)	4.5 (2.3–8)	4 (2–7)
Principal diagnosis category^1^		
- Acute coronary syndrome	34 (13.5%)	136 (13.5)
- Atrial fibrillation	13 (5.2%)	52 (5.2%)
- Chronic heart failure	85 (33.7%)	340 (33.7%)
- Exacerbation of chronic obstructive airways disease	15 (6.0%)	60 (6.0%)
- Other cardiovascular	21 (8.3%)	84 (8.3%)
- Pneumonia	9 (3.6%)	36 (3.6%)
- Thromboembolic	12 (4.8%)	48 (4.8%)
- Other	63 (25.0%)	252 (25.0%)
Married	135 (54.7%)	437 (45.9%)*
Born outside Australia	151 (60.0%)	545 (54.1)
Prior admission in year prior to index	125 (49.6%)	430 (42.7%)
Number of admissions in year prior to index admission (mean ± SD)	1.47 ± 4.68	1.55 ± 5.14
Total LOS for admissions in the year prior to index (mean ± SD)	7.5 ± 14. 8	6.2 ± 15.1*
Discharged to residential care	8 (3.2%)	78 (7.7%)**
Comorbidities during index admission		
- Hypertension	115 (45.6%)	405 (40.1%)
- Diabetes	84 (33.3%)	251 (24.9%)**
- Atrial fibrillation	52 (20.6%)	190 (18.8%)
- Hyperlipidemia	37 (14.7%)	109 (10.8%)
- Chronic heart failure	118 (46.8%)	375 (37.2%)***
- Ischemic heart disease	58 (23.0%)	219 (21.7%)
- Valvular heart disease	13 (5.2%)	66 (6.5%)
- Chronic renal failure	29 (11.5%)	78 (7.7%)
- Obstructive airways disease	33 (13.1%)	146 (14.5%)
- Peripheral vascular disease	6 (2.4%)	24 (2.4%)
- Cerebrovascular disease	9 (3.6%)	53 (5.3%)
- Reflux disease	3 (1.2%)	20 (2.0%)
- Chronic liver disease	5 (2%)	8 (0.8%)
- Depression/anxiety	7 (2.8%)	36 (3.6%)
- Thromboembolic disease	12 (4.8%)	57 (5.7%)
- Dementia	6 (2.4%)	55 (5.5%)*
- Osteoporosis	2 (0.8%)	9 (0.9%)
Total number of comorbid conditions (median and IQR)	2 (1–4)	2 (1–3)*
Proportion of patients with		
- 3 or more comorbid conditions	117 (46.4%)	405 (40.2%)
- 4 or more comorbid conditions	78 (31%)	229 (22.7%)

^1^Item used for matching

Difference between groups: * p<0.05; ** p<0.01; *** p<0.001

### Readmissions and total LOS

A total of 241 (23.9%) in the usual care and 53 (21.0%) in the MACS care had at least one readmission in the follow-up period. The average number of readmissions following discharge from the index admission was 2.08 (± SD 3.45) for patients in the ‘usual care’ group by comparison to patients in the MACS group where it was 2.41 (± SD 4.07). The average total length of stay for these hospitalisations following the index admission was 13.4 days (± SD 27.0) for patients who received usual care and 16.0 days (± SD 33.2) for patients in the MACS group. After adjusting for duration of follow-up and covariates, no significant differences were observed for readmissions or length of stay between the two models of care (Table 2).

**Table 2:** Association of multidisciplinary care model (MACS) with health outcomes following index admission*.

	**Rate Ratio RR (95% CI)**	**p-value**
**Usual Care**	Reference	
**MACS care**		
Number of readmissions		
Unadjusted RR	1.14 (0.88–1.47)	0.31
Adjusted* RR	1.01 (0.79–1.28)	0.95
Total LOS of readmissions		
Unadjusted RR	0.88 (0.60–1.29)	0.52
Adjusted* RR	0.83 (0.56–1.21)	0.32
	**Hazard Ratio HR (95% CI)**	**p-value**
**Usual Care**	Reference	
**MACS care**		
Survival		
Unadjusted HR	0.73 (0.54–0.97)	0.03
Adjusted* HR	0.70 (0.51–0.96)	0.03

*Accounting for total time of follow-up in study and adjusted for age, gender, marital status, country of birth, next of kin proximity, LOS of index admission, number of hospitalisations in the 12 months prior to index admission, primary diagnoses of primary index admission, comorbidities at index admission and discharge destination.

RR, Rate Ratio; HR, Hazard Ratio; LOS, length of stay

### Survival

**Fig 1:**
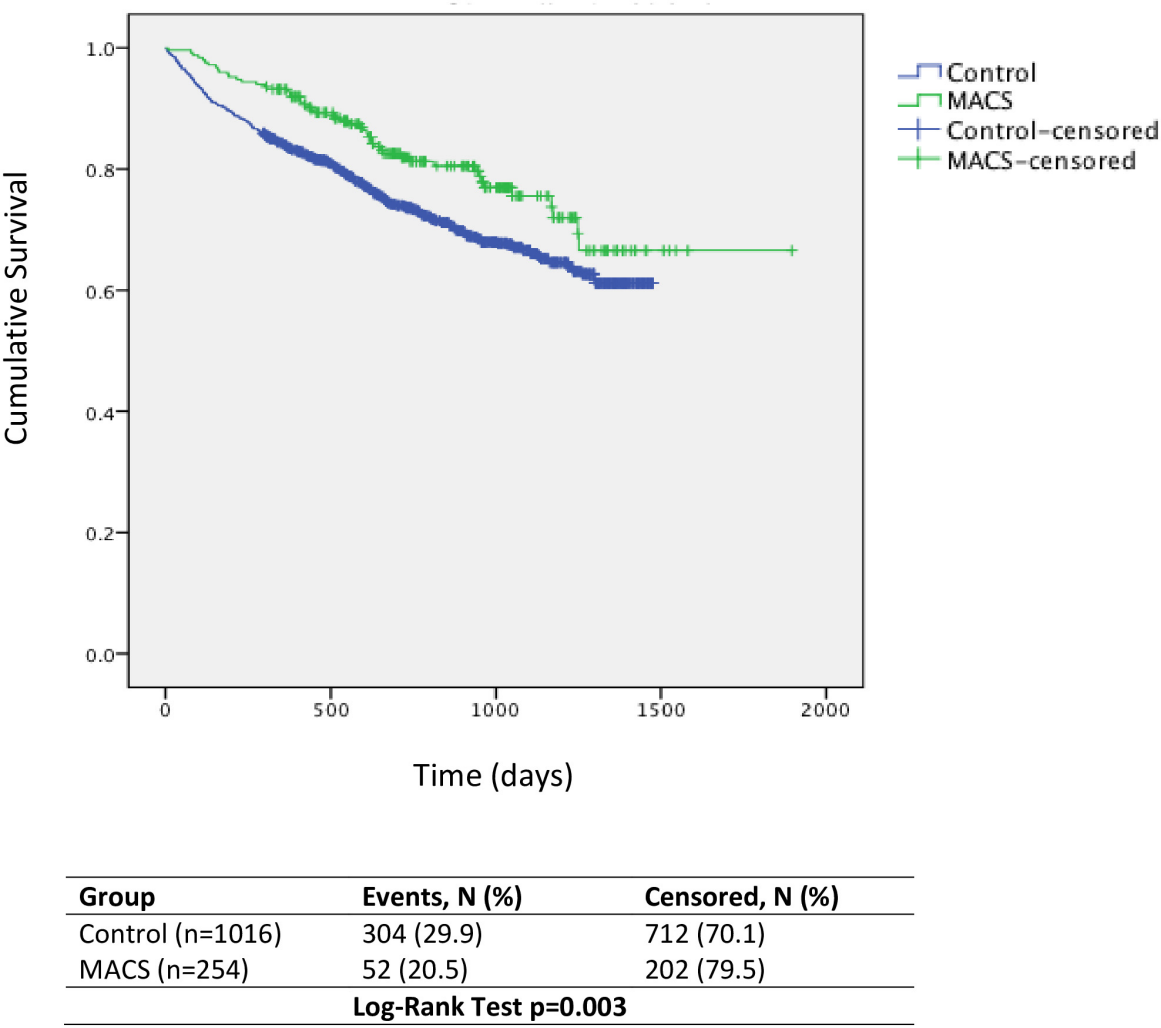
Kaplan Meier Survival Analysis of Time to Death for MACS and Control Groups.

During the study period 29.9% (n = 304) patients who received ‘usual care’ (controls) died, by comparison with 20.6% (n = 52) patients in the MACS group. Kaplan-Meier analysis shows changes in survival between the two groups over the duration of the study (Log-Rank Test p = 0.003) (Fig 1). After adjustment for duration of follow up and covariates, a 30% statistically significantly lower risk of mortality for MACS patients (adjusted HR 0.70 95% CI 0.51–0.96, p = 0.03) was observed compared with control patients (Table 2).

## Discussion

This is the first Australian study to evaluate a holistic multidisciplinary outpatient model of care on readmission, length of stay and mortality in older patients with multiple chronic conditions following discharge from hospital. Despite increased comorbidity (namely chronic heart failure and diabetes) of the patients who received the multidisciplinary care model by comparison to the control patients, this model of care was associated with a significant lower mortality rate.

After adjusting for time of follow up and covariates, a 30% (adjusted HR 0.70 95% CI 0.51–0.96 p = 0.03) reduction in mortality with the MACS model of care was observed by comparison to those patients in the control group who received usual care. The findings of improved survival with the provision of care by a multidisciplinary team is in accord with a previous study which showed a reduction in mortality at two years from 13.2% in the control group to 5.6% in the intervention group (p = 0.02, n = 319).[14] Greater compliance with pharmacological recommendations that are included as key parts of the MACS model of evidence based guideline care [18] that includes initiation and up-titration of ACE inhibitors and beta-blockers in patients with systolic heart failure [19,20] and the use of statins in patients with ischemic heart disease [21] are likely to have contributed to this improved survival. The regular clinic follow up and dial-in service may also have provided more rapid access to health services during disease exacerbations preventing more severe clinical deterioration. The effect size on survival observed in the current study is larger than what may be expected from these factors alone, and referral bias is likely to be a contributing factor. Those with very poor prognosis are unlikely to be referred for ongoing outpatient follow-up and differences in the survival curves between the two groups occurs from onset of follow-up. This referral bias could also be expected to result in greater readmission rates in MACS patients as the service aims to manage patients at high risk of readmission. In accord, the patients included in the MACS model of care had more comorbid conditions, and spent more days in hospital in the year prior to the index admission. Despite this, after adjusting for demographic and clinical factors, MACS patients had similar readmission rates and length of stays. Despite the matching, it would still be anticipated that MACS patients would have a higher readmission rate due to residual confounding by social and functional factors [22,23], disease severity, patient compliance, and other factors which were not adjusted for in the multivariate analysis.

A recent systematic review of interdisciplinary team care interventions on general medical wards, reported that of 23 identified studies, 80% did not reduce readmissions and 70% failed to reduce length of stay. [15] Interestingly, mortality was also largely unaffected by interdisciplinary team care interventions in this review, with the authors suggesting that these typical outcome measures may fail to capture the complexity of the impact of multidisciplinary models of care on patient outcomes. [15] It is likely that in these older more complex patients, length of stay may not be significantly altered without reducing quality of care and similarly the rate of readmissions may not be an appropriate outcome measure, given the likelihood of readmission based on numbers of comorbid conditions. In order to assess the true effect and magnitude of the benefits of multidisciplinary care in terms of readmissions and survival a greater range of patient-specific outcomes that may be better suited to assess the complexities of multidisciplinary care may be required within the context of a randomised controlled trial.

The care provided for the ‘usual-care’ group consisted of general practitioner care, along with multiple specialists managing the various comorbidities through standard outpatient/ambulatory clinics. This approach risks fragmentation of care, with poor communication between care providers, and issues such as social service coordination, lifestyle modification guidance, functional status assessment and end-of-life planning, less likely to be addressed in short, single-condition clinical interventions.[18] There is also evidence of patients finding a single coordinated approach, which can be delivered through a multidisciplinary generalist model, more appealing.[24]

The MACS model involved the systematic implementation of a number of components which have been shown individually to benefit patients with chronic conditions: the initial questionnaire screened for common conditions in those patients with multiple conditions including malnutrition [25], depression [26] and falls[27]. The multidisciplinary team was able to provide care coordination and continuity of care in the transition from hospital to the community [24], disease and self-management education [28,29], and medication reconciliation.[30] Communication with primary care providers was given primacy, with feedback sought and bi-directional partnerships developed in pursuit of mutually agreed goals for each patient.

The population in this study was older and medically complex with a median age of 77 years old, nearly half having had another hospital admission in the preceding year, and approximately 40% having three or more comorbidities. The presence of comorbid conditions can greatly impact on patients’ overall care needs and management strategies. However, as the number of comorbid conditions were determined from the index hospitalisation based on ICD-10 coding from the index admission, it is likely that conditions which did not impact on the hospitalisation but contribute to outpatient patient complexity such as osteoarthritis, osteoporosis, depression, anxiety and cognitive impairment would have been substantially under-estimated.

A limitation of the current study is that it was performed at one tertiary centre and the results may reflect the practice of the individual clinicians rather than the model itself. It is also not possible to know the characteristics of the care received by the usual care group. In addition, as discussed previously, selection / referral bias would likely exaggerate the mortality benefit of the MACS intervention and the two groups may not be truly comparable. A strength of the study is the 4:1 control to cases matching within the same population base (i.e. those admitted to hospital), together with the matching on a number of clinical factors known to be associated with survival and readmission in medical inpatients.

In conclusion, the provision of a holistic multidisciplinary model of care to older comorbid patients post-discharge was associated with significantly improved survival in comparison to patients receiving usual post-discharge care. We propose that further studies that evaluate the benefit of this multidisciplinary care model, with a particular view towards cost-effectiveness, should be conducted.
